# 

**Published:** 1901-12

**Authors:** 


### Society Notes.

### South Texas Medical Association.

HOUSTON, TEXAS, November 1, 1901.

DEAR DOCTOR: Our next meeting will be held in Houston December 12th and 13th. Please let us have a paper from you for this occasion, and send us the title on or before November 30th.

Fraternally,

R. W. KNOX, President.

D. S. WIER, Secretary, Houston.

